# Physical Exercise

**Published:** 1883-01

**Authors:** 


### PHYSICAL EXERCISE.

A lecture under the auspices of the Edinburgh Health Society was lately delivered by Dr. Charles Cathcart, Lecturer on Anatomy in the Edinburgh School of Medicine, on "Physical Exercises; their Place and Function." Dr. Cathcart, after defining physical exercise as to the "action and use of our bones and muscles," went on to explain the anatomy of the muscles, the manner of their attachment to the bones, and their great capacity of contraction—it being stated that the body was arranged into about 400 separate muscles of various sizes and shapes. No muscle could contract unless stimulated by a nervous impulse coming from the brain or spinal cord. Each muscle had its own particular action, but no muscle ever contracted by itself. They could thus see that the exercise of one part of the body indirectly told upon many others which they did not suspect. Hence the value of vigorous walking, for instance, with the swing of the arms, the balance of the body, and the action of the legs; but hence, also, the danger of movements which were one-sided and often repeated, producing the constant and associated action of certain groups of muscles, which might bring about changes in the bones and alterations in form which no one would suspect.

Going on to consider the effect of muscular exercise on the various functions of the body, Dr. Cathcart first noted the changes it caused in the respiration. They were all familiar with the fact that exercise not only made the heart beat quicker, but caused them to breathe more rapidly and freely and at the same time the amounts of carbonic acid and watery vapor exhaled were much increased. Under ordinary circumstances it had been found that a man drew in 480 cubic inches of air per minute; if he walked four miles an hour he drew in 2,400 cubic inches, and if six miles an hour, 3,360 cubic inches. The muscles, in contraction, used more oxygen and gave out more carbonic acid, consequently a greater demand was made on the lungs. More air was required, and the blood must be driven the faster through them; and that accounted for the shortness of breath and beating of the heart which accompanied any muscular action.

If they looked at the demands made upon the air while a person was taking exercise, they would see how very important it was that the air should be not only large in amount, but also exceedingly pure in quality. Let them take, as an example, an ordinary dancing party. There were more people in the room than it was intended for; the whole company exerted themselves violently—certainly as much as would be equal to walking four miles an hour—and what was the consequence? Not only did they now require five times as much air as they did before, but they were using up the oxygen, and giving out the carbonic acid at a relatively much increased proportion—while people were afraid to open the windows in case of draughts. When they remembered that that was almost carried on in a blaze of gas light, every burner of which used as much air as four or five men, they could see that those entertainments required serious attention and careful management if they were to be conducted an sound principles of health.

Emphasizing the point that during exercise the lungs should have the freest possible play, the lecturer said he had been furnished from good authority with an illustration of the effects of custom *versus* humanity and sense in the late Egyptian war. A body of soldiers and another of marines had to make a march of three miles under the burning sun. The soldiers had their tight-fitting jackets, the marines their loose and free costume. Before the march was ended 130 of the soldiers had fallen out, while every man of the sailors continued in his place. He knew of no other difference between the two sets of men but the costume.

The effects of physical exercise on the circulation and on the nervous system were next in turn considered. As to the first as any unwonted strain must act injuriously on the heart, necessity existed for beginning gradually and systematically any exercise which involved unusual exertion. On the latter point, the reasonableness of relaxation and rest to the brain was insisted on, and muscular exertion commended as one of the best cures of mental overwork. That brain and muscle could be developed at the same time was illustrated by a reference to the oarsmen of Oxford and Cambridge; and the intelligence of the working classes was also cited as a proof that hard and constant physical labor in no way tended to depreciate the quality and strength of brain power.

As to the effects of exercise in expanding the chest some striking facts were given, not the least interesting of which related to a school where physical exercise had been systematically carried out. The effect of regular exercise was shown as follows: New boys, age 14, average chest measurement, 29.3 ; at 15, 30.6 ; at 16, 32.0 ; at 17, 32.6 ; and at 18, 32.5 ; while former boys measured respectively 30.6, 32.1, 34.2, 35.8, and 36.8.

In conclusion Dr. Cathcart laid down some rules for regulation of physical exercise : 1. It should be conducted in an abundance of fresh air and in costumes allowing free play to the lungs, and of a material which will absorb the moisture, and which, therefore, should be afterward changed—flannel. 2. There should always be a pleasant variety in the exercise, and an active mental stimulus to give interest at the same time. 3. The exercise should as far as possible involve all parts of the body and both sides equally. 4. When severe in character, the exercises should be begun gradually and pursued systematically, leaving off at first as soon as fatigue is felt. 5. For young people the times of physical and mental work should alternate, and for the former the best part of the day should be selected. 6. Active exertion should be neither immediately before nor immediately after a full meal.

